# Characteristics of amino acids in the surface sediments of the Changjiang Estuary, China

**DOI:** 10.1016/j.dib.2018.05.079

**Published:** 2018-05-23

**Authors:** Kui Wang, Jianfang Chen, Haiyan Jin

**Affiliations:** Key Lab of Marine Ecosystem and Biogeochemistry, Second Institute of Oceanography, State Oceanic Administration, Hangzhou 310012, China

## Abstract

Amino acids (AAs) constitute the largest reservoir of organic nitrogen in most marine organisms and are a major fraction of characterized carbon in marine particulate matter. AAs are also useful indicators of degradation in the marine environment. This article reports original content data of 17 individual amino acids on 47 surface sediment samples and 18 suspended particulate samples in the water column in Changjiang Estuary. Contents of amino acids in suspended particulates were much higher than that in the surface sediments. The data are supplemental to our research paper, entitled “Organic matter degradation in surface sediments of the Changjiang Estuary: Evidence from amino acids” (Wang et al., 2018) [1]. The data are very useful to researchers and policy makers on estuarine benthic environment and ecology.

**Specifications Table**

**Table:** 

Subject area	Chemistry
More specific subject area	Marine chemistry
Type of data	Table
How data was acquired	HPLC Waters 600, Waters corporation.
Data format	Raw, analyzed
Experimental factors	the surface sediments were dried, ground, and acid hydrolyzed
Experimental features	Total hydrolysable amino acids analysis
Data source location	30–32.5°N,121–127°E in the Changjiang estuary
Data accessibility	data are presented in this article

**Value of the data**

• The data presented will be useful for organic matter quality assessments in the Changjiang Estuary and adjacent East China Sea.
• The data will be available for use when comparing the total hydrolyzable amino acid compositions in the sediments of other areas.
• The data will be available to consider the nutrient effect on the benthic animals in the Changjiang Estuary.

## 1 Data

Total hydrolyzable amino acid (THAA) contents of the surface sediments in Changjiang Estuary were measured using a high pressure liquid chromatography [1]. The contents of each type of amino acid are represented in units of mg g^−1^ of sediments dry weight, and the molar percentage of each amino acid relative to total amino acids was also determined. Supplementary Table 1 provides the amino acid contents in the surface sediments collected in April, 2007, and Supplementary Table 2 provides those measured in suspended particulate materials collected in August, 2009.

## 2 Experimental design, materials and methods

### 2.1 Experimental design

Samples of surface sediments (0–2 cm depth; *n*=47) were collected at 47 stations during a research cruise in the Changjiang Estuary, in April 2007. A steel grab sampler was used to recover the sediments, which were subsampled on deck and then frozen at −20 °C for later analysis.

Samples of suspended particulate matter (*n*=18) were collected at 4 stations in August 2009. Each sample of 500–2000 ml seawater was passed through a 47 mm diameter GF/F filter before being stored at −20 °C for later analysis.

### 2.2 Materials and methods

To quantify sedimentary THAA, we dried and ground sediment subsamples before washing them with Milli-Q^®^ water in a supersonic box for desalting. Aqueous hydrolyses were conducted in 6N HCl under N_2_ gas for 24 h at 110 °C. The hydrolysis mixture was dried and then dissolved in Milli-Q^®^ water; the component amino acids were derived according to the Waters^®^ AccQ·Tag^™^ method [2]. The derivatives were determined with a Waters^®^ 600E pump system (multi-solvent delivery system) and a Waters^®^ 474 fluorescence detector; the THAA data were processed with a Waters^®^ Millennium^®32^ chromatography station. Results from duplicate samples of individual AAs showed the coefficient of variation to be <5.6%; for THAA, the coefficient of variation was 2.1%.

For analyses of THAA in suspended particulate matter, samples were collected onto a GF/F membrane and then analyzed according to the same protocol as the surface sediment samples.

The external AA standard solution (Sigma-Aldrich^®^) contained 17 individual amino acids: alanine (Ala), arginine (Arg), aspartic acid (Asp), cystine (Cys), glutamic acid (Glu), glycine (Gly), histidine (His), isoleucine (Iso), leucine (Leu), lysine (Lys), methionine (Met), phenylalanine (Phe), proline (Pro), serine (Ser), threonine (Thr), tyrosine (Tyr), and valine (Val). Two non-protein amino acids, β-alanine (β-Ala) and γ-amino butyric acid (γ-Aba) were added to this solution (Supplementary Tables 1 and 2).
